# Intercropping of Green Garlic (*Allium sativum* L.) Induces Nutrient Concentration Changes in the Soil and Plants in Continuously Cropped Cucumber (*Cucumis sativus* L.) in a Plastic Tunnel

**DOI:** 10.1371/journal.pone.0062173

**Published:** 2013-04-24

**Authors:** Xuemei Xiao, Zhihui Cheng, Huanwen Meng, Lihong Liu, Hezi Li, Yinxin Dong

**Affiliations:** College of Horticulture, Northwest A&F University, Yangling, Shaanxi, China; United States Department of Agriculture, United States of America

## Abstract

A pot-based experiment was conducted to investigate nutrient concentrations in cucumber plants intercropped with various amounts of green garlic. In addition, the soil nutrient contents were studied over two consecutive growing seasons. The results revealed that the accumulation of biomass and the nutritional elements nitrogen (N), phosphorus (P), potassium (K), calcium (Ca) and manganese (Mn) in cucumber plants were significantly increased for intercropping treatments during the two growing seasons compared to monoculture. Conversely, magnesium (Mg) concentrations were decreased in the cucumber plants. Shoot iron (Fe) concentrations decreased whereas root Fe concentrations increased in the intercropping system. Shoot and root zinc (Zn) concentrations decreased during the fall of 2011 but increased during the spring of 2012. Soil organic matter and available N, P and K were significantly increased as the proportion of intercropped green garlic increasing. Medium levels of intercropping green garlic improved cucumber nutrient concentrations the most. The regression analysis showed that the concentrations of most elements were significantly related to the amounts of garlic bulbs, especially the microelements in the spring 2011. The available soil N and organic matter were linearly related to the amounts of garlic bulbs. The results indicate that the nutritional status of the soil and plants of continuously cropped cucumber could be improved by intercropping with green garlic.

## Introduction

Cucumber **(** *Cucumis sativus* L.**)** is commonly cultivated in continuous cropping monoculture under protected cultivation, resulting in poor growth, reduced crop yield and “soil sickness” [1]–[3]. Soil sickness may involve nutrient uptake disorders and changes in the availability of soil nutrients [4]–[5], adverse effects on soil structure [6]–[7] and the build-up of soil-borne diseases and autotoxicity [8]–[9].

Intercropping is becoming more important in improving the utilization of land resources and increasing crop productivity [10]–[11]. Some studies have demonstrated that intercropping can relieve soil sickness by improving soil quality [10] [12] and the ecological microclimate [13]. In addition, intercropping can effectively improve nutrient mobilization in the rhizosphere and nutrient acquisition based on inter-specific root interactions [14]–[15]. Most studies on nutrient uptake and transfer have focused on legume-cereal intercropping systems [16]–[19]. Nitrogen (N) input and transfer occurs in this intercropping system based on the specific biological nitrogen fixation of leguminous crops [16] [18]. Intercropping with legumes also facilitates phosphorus (P) uptake and utilization in cereals [20]–[22]. The use of a P source can improve calcium (Ca) and magnesium (Mg) uptake due to rhizosphere acidification [23]. The uptake and mobilization of micronutrients is also influenced by intercropping, especially Fe in peanut and maize intercropping systems [15] [24]. Except for legume-cereal intercropping systems, other studies reported that the turmeric, maize and onion intercropping systems improved nutrient uptake compared to that of either of the sole crops [25]. Moreover, Shanmugham [26] reported that intercropping of onion improved P uptake of cotton based on chelating compounds exuded by onion increasing the available P to cotton plants (cited from [11]).

Garlic **(** *Allium sativum* L.) has effectively been incorporated into an intercropping system as a companion crop due to its allelopathic and antimicrobial effects. Some studies have reported that garlic intercropping can prevent insect attack [27]–[28] and weed invasion [29]. Recent research demonstrated that intercropping with garlic, green garlic (planting whole bulbs and cutting green garlic more than once) [30] and onion [31] can alleviate problems associated with continuous cropping (soil sickness) of cucumbers under protected cultivation. However, most attention has been directed to the yield and growth parameters, as well as soil biological characteristics. The objective of this study was to ascertain the characteristics of nutrient uptake and utilization in a continuously cropped cucumber crop intercropped with green garlic. It has been reported that garlic (shoots, roots and bulbs) exhibits a “hormesis effect” at increased concentrations [32]–[33]. For this reason, various levels of intercropping with green garlic were tested in this study to select the best concentration and investigate their effects on the N, P, K, Ca, Mg, Fe, Mn, Zn concentrations of cucumber plants and changes in the levels of soil organic carbon and available N, P and K in this intercropping system.

## Materials and Methods

### Growth Medium and Experimental Design

The pot-based experiment was conducted from fall 2011 to spring 2012 in a plastic tunnel at the experimental station (Northwest China, N 34° 16', E 108° 4') of the College of Horticulture, Northwest A&F University, in Yangling, Shaanxi province, China. Pot soil was collected from another plastic tunnel, which had been previously planted with cucumber for five years. The chemical characteristics of the soil were as follows: electrolytic conductivity (1∶5 soil: water) 340 µS·cm^−1^; pH (1∶1 soil: water) 7.59; organic matter 25.82 g·kg^−1^; total N 2.15 g·kg^−1^; available N 110.13 mg·kg^−1^; total P 1.34 g·kg^−1^; available K 216.32 mg·kg^−1^ and available P 191.61 mg·kg^−1^. Seventeen kilograms of soil were put into each plastic pot (46 cm diameter ×40 cm depth). The pots were arranged in a complete randomized block design with three replicates for each treatment. Ten pots were used for each treatment in one replication.

The experiment was designed to test one factor (the amount of intercropped green garlic) at the following six levels: 0, 150, 300, 450, 600 and 750 g of garlic bulbs (which were planted with the intention of repeatedly harvesting green garlic). Monocropped cucumber was used as a control.

### Experimental Arrangement

In northern China, cucumber is often planted two cropping in plastic tunnel, namely spring cucumber and fall cucumber, respectively. In our experiment green garlic is interplanted with fall cucumber in early fall, cut harvested for about three times during fall and early winter, then totally removed in spring of next year before cucumber is transplanted.

In fall 2011, before the fall cucumbers were transplanted, experimental pots were uniformly tilled by hand and fertilized with 100 g of organic fertilizer, 10 g of calcium superphosphate and 10 g of compound fertilizer (N-P_2_O_5_-K_2_O:18-18-18) per pot. Cucumber seedlings (Xintiandi No. 1) were purchased from the Xintiandi Agriculture Science and Technology Demonstration Garden at Yangling and transplanted on August 13. Each cucumber seedling was planted in the centre of a pot. Twenty days later, various amounts of garlic bulbs were uniformly planted around the cucumber plants. The cucumbers and the garlic bulbs were planted 10–12 cm apart. After 45 days of co-growth, the fall cucumber plants were totally removed from pots at the full bearing stage. Green garlic was growing until the spring of 2012 and removed on March 19. All pots were fertilized according to the previous growing season. On March 21, spring cucumber seedlings were transplanted and grown for one and a half months and then harvested at the early fruiting stage. During the experiment, soil moisture was maintained at approximately 70% of the field water-holding capacity. All pots were irrigated well manually and hand-weeded during crop growth.

### Biomass and Plant Nutrient Element Analysis

At harvest, cucumber plant samples were divided into shoots and roots and weighed for fresh weight determinations. The samples were then rinsed with tap water and distilled water. The dry weights of the shoots and roots were measured after drying at 65°C for 72 h to a constant weight after killing enzymatic activity at 105°C for 15 min. The samples (shoots and roots) were pulverized to pass through 0.149 and 0.250 mm mesh screens and analyzed to determine the total N, P, K, Ca, Mg, Fe, Zn and Mn contents. To analyze N, P and K, the samples were digested a mixture of concentrated H_2_SO_4_ and H_2_O_2_. The nitrogen (N) content was analyzed using a semi-micro Kjeldahl apparatus [34]. The phosphorus (P) content was evaluated spectrophotometrically, and the potassium (K) content was measured by an atomic absorption spectrophotometer (Hitachi Z-2000, Tokyo). Prior to measuring of the remaining nutrients, the samples were subjected to dry ashing at 550°C for 6 h and then dissolved (1∶1, v*/*v) in 5 mL of hydrochloric acid (HCl) and finally diluted to 25 mL with deionized water. The extracts were then filtered and stored in plastic vials until analyzed. Ca, Mg, Fe, Zn and Mn were determined using an atomic absorption spectrophotometer (Hitachi Z-2000, Tokyo).

### Soil Analysis

Soil taken from a depth of 0–10 cm was sampled from each of the ten pots randomly at cucumber harvest. Cucumber roots were taken from the pots and gently shaken by hand to obtain soil that had adhered to the roots, hereafter referred to as rhizosphere soil. Soil that was more distant from the roots was regarded as bulk soil [35]. After air-drying, the bulk soil samples were pulverized and passed through 1.000 and 0.149 mm sieves for analysis of organic carbon, available N, available P and available K. Rhizosphere soil were pulverized and passed through 1.000 mm sieves for analysis of pH. Rhizosphere soil pH was prepared by a 1∶1 water extract and measured using a pH meter. Soil organic carbon was estimated using the dilution heat K_2_Cr_2_O_7_ oxidation volumetric method. Available N was analyzed through the alkali-hydrolytic diffusion method [36]. Available P was determined by extraction with 1 M sodium bicarbonate (NaHCO_3_) according to the method of Olsen et al. [37]. Air-dried soil samples were extracted with 1 M ammonium acetate (NH_4_Ac), and the available K concentration of the filtrate was determined using atomic absorption spectrophotometer (Hitachi Z-2000, Tokyo).

### Statistical Analysis

Statistical analysis was carried out using SAS 8.1 software. Data were analyzed by ANOVA for a completely randomized block design. The difference of the treatments was compared using the least significant difference (LSD) test at *P*≤0.05, *P*≤0.01 and *P*≤0.001. Simple regression (REG) analysis was used to examine relationships between the amounts of garlic bulbs and biomass, nutrient concentrations in cucumber plants, available soil N, P, K and organic carbon content.

## Results

### The Effect of Intercropping with Green Garlic on the Biomass of Continuously Cropped Cucumber in an Experiment Carried out in Fall 2011 and Spring 2012

**Figure 1:**
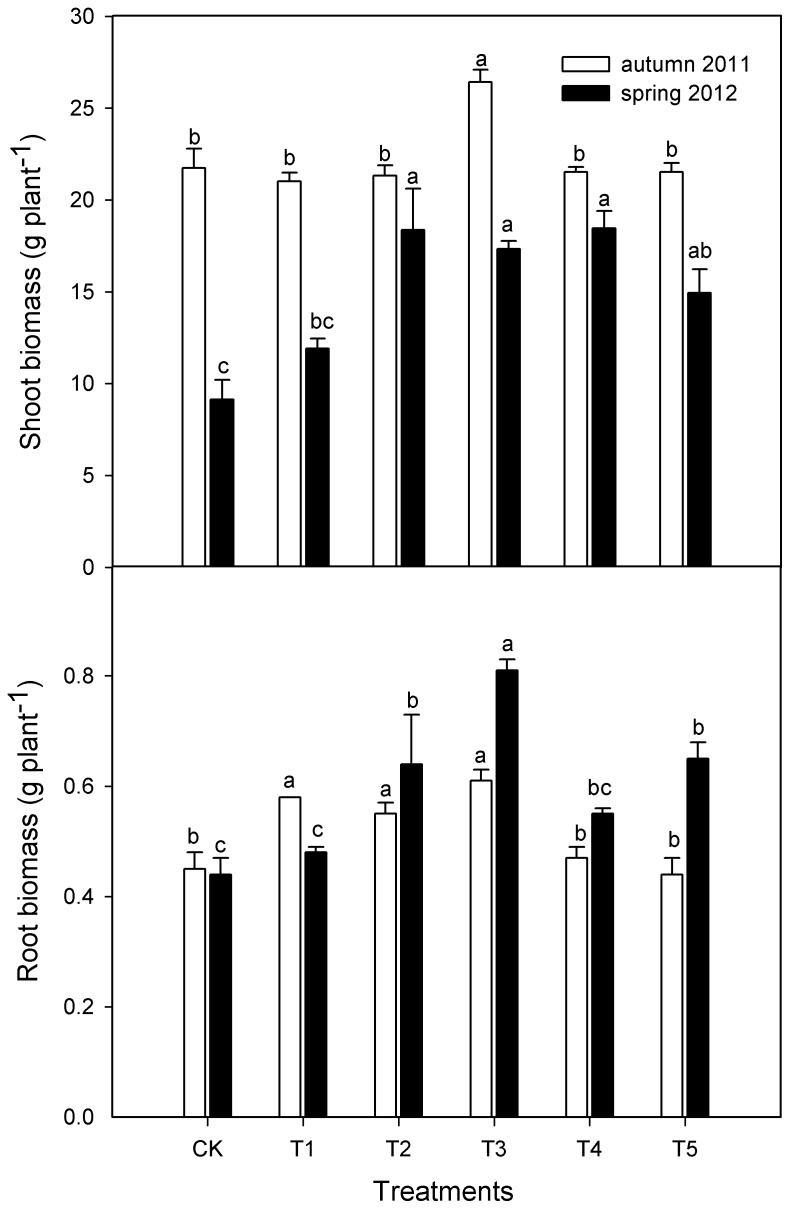
Cucumber shoot and root biomasses with various amounts of intercropped green garlic in the fall of 2011 and the spring of 2012. Samples were taken 45 days after cucumber intercropping with green garlic in the fall of 2011 and the transplantation of cucumber in the spring of 2012. CK represents cucumber monocropping. T1, T2, T3, T4 and T5 represent 150, 300, 450, 600 and 750 g of garlic bulbs intercropped with cucumber, respectively. Different letters above bars indicate significant differences at the P<0.05 level, n = 10.

The shoot and root biomass of cucumber was significantly increased by intercropping with green garlic during two growing seasons (Figure 1). Only treatment T3 (intercropping with 450 g of bulbs for green garlic production) exhibited a significant positive effect on shoot biomass during the fall of 2011. With the exception of treatment T1 (intercropping with 150 g of bulbs for green garlic production), intercropping with green garlic significantly increased shoot biomass until the spring of 2012. And shoot biomass was significantly related to the amounts of garlic bulbs (Y = 8.4832+0.04046X-0.00004185X^2^, R^2^ = 0.9024). Otherwise, cucumber root biomass was more susceptible to intercropped green garlic; cucumber root biomass was significantly promoted by relatively small amounts (150, 300 and 450 g) of bulbs grown for green garlic production during the first growing season. In contrast, relatively large amounts (300, 450 and 750 g) exhibited the same effect on root biomass during the spring of 2012.

### The Effect of Intercropping with Green Garlic on Plant Nutrient Concentrations in Continuously Cropped Cucumber during the Experiment Carried out in Fall 2011

The co-growth stage of cucumber intercropped with green garlic lasted for 45 days in fall 2011, and the concentrations of nutrient elements in the cucumber plants were significantly affected to varying degrees by the intercropping of green garlic at different bulb-planting rates (Table 1 and 2). Except for the shoot concentration of nitrogen (N), the macroelement contents of the cucumber plants were significantly influenced (Table 1). Intercropping with any amount of green garlic except for 150 g (T1) significantly increased the N concentration of cucumber roots and the P concentration in the shoots. P concentrations of shoot were significantly linearly related to the amounts of garlic bulbs (Y = 0.36048+0.0001809X, r^2^ = 0.6617). Only treatment T2 (intercropping with 300 g of bulbs for green garlic production) exhibited a significantly stimulative effect on root P concentrations. The concentrations of K in the shoots and roots were significantly promoted by treatment T4 (intercropping with 600 g of bulbs for green garlic production). Shoot calcium (Ca) concentrations exhibited an up and down trend with increasing amounts of green garlic. Treatments T4 and T5 significantly increased root calcium (Ca) concentrations, which were significantly linearly related to the amounts of garlic bulbs (Y = 19.1348+0.001410X, r^2^ = 0.7609). No significant difference in shoot magnesium (Mg) concentrations was observed among the treatments. Surprisingly, root Mg concentrations were significantly reduced by any amount of intercropping with green garlic.

**Table 1:** The effect of various amounts of intercropped green garlic on the macronutrient concentrations of cucumber plants in the experiment carried out during the fall of 2011.

Treatment	Amount of bulbs intercropped (g)	N (%)		P (%)		K (%)		Ca (mg g-1)		Mg (mg g-1)	
		shoot	root	shoot	root	shoot	root	shoot	root	shoot	root
CK	0	4.75±0.08	3.15±0.01	0.36±0.01	0.98±0.01	2.20±0.03	2.84±0.06	20.07±0.17	18.84±0.20	4.58±0.08	10.18±0.16
T1	150	4.58±0.09	3.11±0.01	0.37±0.01	0.96±0.01	2.23±0.06	3.16±0.02**	22.27±0.28**	19.73±0.15	4.50±0.38	8.08±0.30***
T2	300	5.11±0.01	3.51±0.03***	0.45±0.01***	1.06±0.01***	2.09±0.01	2.78±0.07	21.32±0.81	19.57±0.29	4.78±0.14	7.60±0.08***
T3	450	4.53±0.26	3.37±0.01***	0.40±0.01*	0.95±0.01	2.60±0.04***	2.81±0.05	20.77±0.09	19.70±0.21	4.25±0.14	8.75±0.43**
T4	600	4.70±0.09	3.45±0.04***	0.52±0.00***	0.96±0.01	2.50±0.05***	2.91±0.09	17.84±0.72**	20.02±0.12*	4.77±0.27	8.08±0.08***
T5	750	4.60±0.08	3.26±0.01**	0.47±0.01***	0.98±0.00	2.22±0.02	2.96±0.02	18.13±0.26*	20.12±0.59*	4.03±0.12	8.22±0.37***

Values are the means ± standard error of three replicates.

* Significant at the 0.05 probability level.

** Significant at the 0.01 probability level.

*** Significant at the 0.001 probability level.

**Table 2:** The effect of various amounts of intercropped green garlic on the micronutrient concentrations of cucumber plants in the experiment carried out during the fall of 2011.

Treatment	Amount of bulbs intercropped (g)	Fe (µg g-1)		Mn (µg g-1)		Zn (µg g-1)	
		shoot	root	shoot	root	shoot	root
CK	0	186.67±2.85	1390.55±7.83	32.83±1.36	48.92±0.71	43.00±0.87	49.79±0.63
T1	150	166.00±8.72	1501.67±56.45	34.67±0.44	48.50±2.60	37.17±1.01**	47.00±1.26
T2	300	203.28±10.22	1288.22±77.37	34.46±0.84	47.12±1.42	34.18±1.41***	46.82±1.19
T3	450	128.67±4.63***	1285.00±29.30	32.17±0.17	46.83±1.69	43.50±0.58	45.83±1.48*
T4	600	146.19±4.21**	1350.63±4.72	30.62±0.64	48.11±2.40	44.31±0.65	39.73±0.87***
T5	750	162.97±8.48*	1993.53±5.96***	33.33±0.93	68.01±1.45***	41.83±1.30	40.20±1.46***

Values are the means ± standard error of three replicates.

* Significant at the 0.05 probability level.

** Significant at the 0.01 probability level.

*** Significant at the 0.001 probability level.

The microelement contents were also significantly affected by intercropping with green garlic (Table 2). Intercropping with relatively large amounts (450 g, 600 g and 750 g) of bulbs (for green garlic production) significantly decreased shoot iron (Fe) concentrations in cucumber. In contrast, root concentrations of Fe were significantly increased using treatment T4, as did root manganese (Mn) concentrations. Intercropping with relatively small amounts (150 and 300 g) and large amounts (450, 600 and 750 g) of bulbs showed significantly negative effects on shoot and root concentrations of Zn, respectively. Root Zn concentrations were significantly linearly related to the amounts of garlic bulbs (Y = 49.9486−0.01348X, r^2^ = 0.8753).

### The Effect of Intercropping with Green Garlic on Plant Nutrient Concentrations in Continuously Cropped Cucumber during the Experiment Carried out in Spring 2012

Although the green garlic was harvested before the cucumbers were transplanted in spring, the effect of the garlic on nutrient uptake existed until 45 days after cucumber transplantation in spring 2012. Intercropping with various amounts of green garlic displayed significantly different effects on the nutrient element contents (Table 3 and 4). The shoot and root concentrations of N and P in the cucumbers were significantly increased in all treatments compared with monocropping (Table 3). Shoot P concentrations were significantly related to the amounts of garlic bulbs (Y = 0.5057+0.0005733X−0.00000006984X^2^, R^2^ = 0.9489), and root P showed the same trend (Y = 0.5293+0.0007452X−0.00000007778X^2^, R^2^ = 0.8814). Intercropping with relatively small amounts (150 and 300 g) of bulbs significantly promoted shoot whereas large amounts (450, 600 and 750 g) did the same for root K concentrations, which were significantly related to the amounts of garlic bulbs (Y = 5.2679−0.0003929X+0.000001480X^2^, R^2^ = 0.8961). Shoot and root Ca concentrations were significantly enhanced by intercropping with 150 g (T1) and 600 g and 750 g (T4 and T5) of bulbs, respectively. Except for T1, shoot Mg concentrations were significantly decreased. The change of Mg concentrations in shoot was significantly related to the amounts of garlic bulbs (Y = 13.4354−0.01146X+0.000008900X^2^, R^2^ = 0.9652). No significant difference was found in root Mg concentrations among the treatments.

**Table 3:** The effect of various amounts of intercropped green garlic on the macronutrient concentrations of cucumber plants in the experiment carried out during the spring of 2012.

Treatment	Amount of bulbs intercropped (g)	N (%)		P (%)		K (%)		Ca (mg g-1)		Mg (mg g-1)	
		shoot	root	shoot	root	shoot	root	shoot	root	shoot	root
CK	0	3.71±0.06	2.90±0.04	0.50±0.01	0.55±0.01	4.11±0.06	5.26±0.02	54.55±4.02	21.10±0.97	13.50±0.58	9.08±0.22
T1	150	4.44±0.01***	3.47±0.05***	0.59±0.01***	0.59±0.01*	4.90±0.09***	5.25±0.04	65.42±6.32*	22.90±1.06	12.00±1.04	8.67±0.58
T2	300	4.26±0.06***	3.12±0.03**	0.61±0.00***	0.67±0.01***	4.68±0.02***	5.26±0.04	55.33±2.32	22.73±0.38	10.33±0.73*	8.83±0.36
T3	450	4.11±0.02**	3.55±0.10***	0.61±0.01***	0.74±0.01***	4.12±0.06	5.49±0.02***	56.33±0.98	21.67±0.52	10.33±0.44*	9.17±0.46
T4	600	4.27±0.02***	3.33±0.02***	0.61±0.00***	0.70±0.01***	4.06±0.06	5.44±0.04**	53.42±2.32	24.13±0.85*	10.00±1.00**	8.75±0.14
T5	750	4.03±0.17*	3.52±0.03***	0.54±0.01*	0.64±0.00***	4.14±0.05	5.85±0.00***	53.58±1.21	24.80±0.97**	9.67±0.73**	8.58±0.44

Values are the means ± standard error of three replicates.

* Significant at the 0.05 probability level.

** Significant at the 0.01 probability level.

*** Significant at the 0.001 probability level.

**Table 4:** The effect of various amounts of intercropped green garlic on the micronutrient concentrations of cucumber plants in the experiment carried out during the spring of 2012.

Treatment	Amount of bulbs intercropped (g)	Fe (µg g-1)		Mn (µg g-1)		Zn (µg g-1)	
		shoot	root	shoot	root	shoot	root
CK	0	370.00±15.28	538.33±30.05	61.67±0.60	27.00±1.04	44.67±0.33	60.33±1.76
T1	150	313.67±4.70***	1351.67±59.47***	61.50±0.76	35.50±1.89***	48.00±0.58	64.33±2.96
T2	300	329.67±3.18**	1380.00±39.69***	64.83±0.73*	48.67±0.73***	50.00±2.65	70.00±0.58**
T3	450	338.33±6.17**	1401.67±47.64***	70.33±0.44***	51.33±1.88***	51.00±2.00*	77.00±2.00***
T4	600	289.00±3.21***	943.33±36.55***	61.33±0.33	42.50±1.50***	50.33±1.86*	77.33±1.20***
T5	750	299.33±2.33***	648.33±6.01	61.33±1.59	32.67±0.17*	51.33±1.76*	75.33±1.76***

Values are the means ± standard error of three replicates.

* Significant at the 0.05 probability level.

** Significant at the 0.01 probability level.

*** Significant at the 0.001 probability level.

As is shown in Table 4, intercropped green garlic significantly lowered Fe concentrations in shoot, while facilitated in root. Root Fe concentrations were significantly related to the amounts of garlic bulbs (Y = 644.8193+4.3329X−0.005940X^2^, R^2^ = 0.8889). Compared to monocropping cucumber, intercropping with medium amount (300 g, 450 g) of bulbs significantly increased the Mn concentrations of whole plant. Root Mn concentrations were significantly related to the amounts of garlic bulbs (Y = 25.2032+0.1168X−0.0001426X^2^, R^2^ = 0.9358). As the amount increasing, intercropping with green garlic enhanced significantly Zn concentrations in shoot and root. The regression analysis showed that both shoot and root Zn concentrations were significantly related to the amounts of garlic bulbs (Y = 44.9432+0.02116X−0.00001772X^2^, R^2^ = 0.9570; Y = 59.0200+0.05362X−0.00004076X^2^, R^2^ = 0.9483).

### The Effect of Intercropping with Green Garlic on Soil Nutrient Characters in Continuously Cropped Cucumber during the Experiments Carried out during Fall 2011 and Spring 2012

The soil nutrients and cucumber rhizosphere pH values were significantly affected by intercropping with green garlic (Tables 5 and 6). Levels of available N, P and K in the soil were higher for the intercropped treatments, especially when relatively large amounts of green garlic were intercropped, compared to the monocropped cucumber cops in both growing seasons. Moreover, intercropping with green garlic increased the available levels of K as reflected in the N: P: K ratio. Available soil N was significantly linearly related to the amounts of garlic bulbs in both fall 2011 and spring 2012 (Y = 109.4048+0.04008X, r^2^ = 0.7780; Y = 114.0571+0.01745X, r^2^ = 0.7976). During both growing seasons, intercropping with green garlic significantly increased the soil organic carbon content. Moreover, the most amount of green garlic exhibited the highest content of soil organic carbon. Only in the fall of 2011, soil organic carbon content was significantly related to amounts of garlic bulbs (Y = 13.8452+0.004320X, r2 = 0.8429). The pH of cucumber rhizosphere soil was not significantly affected by intercropping with green garlic during the fall of 2011 (Table 5). However, in spring 2012, the pH of cucumber rhizosphere soil was lower in the intercropping system compared that in monocropped cucumber (Table 6).

**Table 5:** The effect of various amounts of intercropped green garlic on available soil N, P and K, organic carbon and rhizosphere pH in the experiment carried out during the fall of 2011.

Treatment	Amount of intercropped bulbs (g)	Available soil N (mg kg^−1^)	Available soil P (mg kg^−1^)	Available soil K (mg kg^−1^)	Soil organic carbon (g kg^−1^)	Rhizosphere pH (1∶1 soil: water)
CK	0	110.8±5.8	185.8±2.8	285.1±5.9	13.95±0.09	7.30±0.01
T1	150	110.8±7.7	224.0±9.4**	420.8±2.4***	14.45±0.07	7.28±0.03
T2	300	128.3±5.8	237.1±9.8***	399.0±5.3***	15.20±0.28**	7.25±0.03
T3	450	119.6±7.7	221.9±7.9**	427.9±4.6***	16.01±0.27***	7.24±0.02
T4	600	140.0±5.1**	234.0±8.4**	520.8±2.9***	15.48±0.29**	7.29±0.03
T5	750	137.1±2.9**	212.3±7.7*	437.2±3.3***	17.71±0.41***	7.35±0.02

Values are the means ± standard error of three replicates.

* Significant at the 0.05 probability level.

** Significant at the 0.01 probability level.

*** Significant at the 0.001 probability level.

**Table 6:** The effect of various amounts of intercropped green garlic on available soil N, P and K, organic carbon and rhizosphere pH in the experiment carried out during the spring of 2012.

Treatment	Amount of intercropped bulbs (g)	Available soil N (mg kg^−1^)	Available soil P (mg kg^−1^)	Available soil K (mg kg^−1^)	Soil organic carbon (g kg^−1^)	Rhizosphere pH (1∶1 soil: water)
CK	0	115.7±1.7	186.2±9.4	287.2±5.9	14.77±0.08	7.66±0.02
T1	150	116.2±1.1	190.6±1.1	388.9±0.3***	16.91±0.07***	7.32±0.02***
T2	300	122.5±0.4*	216.8±5.8*	348.5±1.4***	16.96±0.25***	7.53±0.02**
T3	450	128.1±2.5**	190.6±5.3	329.6±1.1***	16.99±0.09***	7.37±0.02***
T4	600	126.2±2.0**	226.8±11.9**	375.6±0.9***	17.34±0.09***	7.27±0.04***
T5	750	126.9±3.6**	214.7±11.9*	407.8±1.5***	17.51±0.09***	7.43±0.01***

Values are the means ± standard error of three replicates.

* Significant at the 0.05 probability level.

** Significant at the 0.01 probability level.

*** Significant at the 0.001 probability level.

## Discussion

This study demonstrated that biomass and the concentrations of N, P, K, Ca and Mn in cucumber plants were increased by intercropping with green garlic during the two studied growing seasons (Figure 1 and Tables 1, 2, 3, 4). The increased concentrations of these nutrients could be related to the enhancement of soil organic matter and available nutrients (Table 5 and 6). There might be two reasons for the increase in soil organic matter in the intercropping system. Garlic bulbs (used to grow green garlic) with their abundant nutrients increased the input of nutrient compared to the cucumber monocrop. Plant residues as one of major fractions of soil organic matter may be present in various stages of decomposition [38] **.** So on the other hand, the green garlic produced numerous roots, which may be remained as a carbon source in the soil after harvesting in the spring of 2012. Some studies have shown that intercropping with garlic was able to improve soil enzyme activities, and the effects lasted to the second growing season [30]–[31]. Soil enzyme activities can be used as suitable indicators of soil quality [39] and play a crucial role in the decomposition of organic residues and nutrient cycling in the soil [40]. In the present study, the observed increase in available N, P and K in the soil could be attributed to higher soil enzyme activities in the green garlic and cucumber intercropping system [30]. The level of available K (as reflected in the N: P: K ratio) was increased in the intercropping systems, which better reflects the nutritional requirements of cucumbers during the fruiting period, when they were sampled. This finding indicates that the soil nutrient balance in the continuously cropped cucumber system was enhanced by intercropping with green garlic. From fall 2011 to spring 2012, available N, P and K levels in the soil increased in the control treatment but decreased in the intercropped treatments, indicating that intercropping with green garlic improved nutrient uptake in the continuously cropped cucumbers even though the garlic was harvested before the cucumbers were transplanted.

Large ion antagonistic effects have been reported between K, Ca and Mg in some plants [41]–[42]. In our study, Mg concentrations were decreased by intercropping with green garlic whereas K and Ca contents were increased. In the fall of 2011, the lower Zn concentrations in cucumber plants may be partly due to an increase in P availability [43]–[44]. However, Zn concentrations were significantly increased by intercropping with green garlic in the spring of 2012, which was related to the lower pH of cucumber rhizosphere soil (Table 6). This is supported by the findings of a report [45] that a decrease in soil pH could enhance the availability of some nutrients in soil, particularly Fe, Mn and Zn. Most studies of Fe uptake have focused on graminaceous plants and dicotyledon intercropping systems. Two mechanisms are present, including increases in the ferric reductase activity of roots and rhizosphere acidification as “Strategy I” [46]–[47] by dicotyledons and exuding phytosiderophores to increase the available Fe by graminaceous plants [48]. Incredibly, the present study showed that Fe concentrations were decreased in the shoots of cucumbers but increased in the roots in the intercropping system. This result indicates that intercropping of green garlic inhibits the transfer of Fe from the roots to the shoots in cucumber plants. However, the available data and literature could not provide an explanation of the mechanisms involved.

In the present study, although the available nutrients and organic matter increased in the soil with increasing amounts of green garlic, the highest nutrient uptake by the cucumber plants did not always result from the highest amounts of green garlic. The result may be ascribed to the allelopathic feature of garlic, which exhibits a positive effect at low concentrations and a negative effect at high concentrations by bioassay [33]. However, further research into the mechanism behind the effect of green garlic on nutrient uptake in cucumbers is needed, particularly with regard to the aspect of root exudates.

## Conclusion

This study indicates that intercropping with green garlic significantly affects nutrient concentrations and the soil nutrient character in cucumber crops. The accumulation of biomass and most nutrient elements (N, P, K, Ca and Mn) in intercropped cucumber was significantly greater than that found in monocropped cucumber, and the effect was even sustained to the second growing season. Mg concentrations were decreased by intercropping. Fe concentrations in cucumber shoots were decreased whereas those in the roots increased in the intercropping system. In the fall of 2011, Zn concentrations were decreased, but these increased in the second growing season. The best effects were always found when medium amounts (300 g, 450 g, 600 g) of bulbs were planted for green garlic production. Soil organic matter and available N, P and K were higher in the green garlic-cucumber intercropping system than in a cucumber monocropping system. The regression analysis showed that the concentrations of the most elements were significantly related to the amounts of garlic bulbs, especially the microelements in the experiment of spring 2011. The available soil N and organic matter were linearly related to the amounts of garlic bulbs.
